# Physician explanation of Z-coded homelessness in medicaid claims

**DOI:** 10.1007/s10742-025-00360-x

**Published:** 2025-09-15

**Authors:** Michael Enich, Emmy Tiderington, Joel C. Cantor

**Affiliations:** 1 Internal Medicine Residency Program, University of Washington, 1969 NE Pacific St, Seattle, WA 98195 USA; 2 School of Social Work, Rutgers, The State University of New Jersey, 390 George St, New Brunswick, NJ 08901 USA; 3 Center for State Health Policy, Institute for Health, Health Care Policy and Aging Research, Rutgers, The State University of New Jersey, 112 Paterson St., 5th floor, New Brunswick, NJ 08901 USA; 4 Edward J. Bloustein School of Planning and Public Policy, Rutgers, The State University of New Jersey, 33 Livingston Ave, New Brunswick, NJ 08901 USA

**Keywords:** Homelessness, Social determinants of health, Z codes, Health-related social needs, Population health

## Abstract

The International Classification of Diseases, 10th Revision (ICD-10) introduced a set of social determinants of health (SDOH) codes including Z59.0, indicating homelessness. Z codes are not widely used, and it is not clear how Z59.0 is used to document homelessness. The goal of this study was to examine patterns of Z59.0 within a linked administrative data set and explore physician explanations for coding prevalence. This study used sequential explanatory mixed methods, first examining claims data from New Jersey Medicaid linked to Homeless Management Information System (HMIS) data for adults aged 18 and older in 19 in 21 counties from 2014 to 2016 (*n* = 724,463). The rate and patterns of Z59.0 coding was compared to HMIS-recorded homeless service use. Then we conducted semi-structured interviews with 18 physicians at high-coding hospitals analyzed via thematic analysis. Only 1.1% of inpatient and ED claims were Z-coded. Claims for male individuals who were age 43–59, Non-Hispanic/White, enrolled via Medicaid expansion, of higher health burden, in the inpatient setting, or chronically homeless were more frequently Z-coded; this was consistent with physician expectations. Physicians were surprised by the frequency Z-coding of claims for individuals who were Non-Hispanic/White and could not give clear explanations as to why some hospitals had higher Z code prevalence. They suggested that individuals who had a Z code without known homeless service use *were* experiencing homelessness. This study suggests Z-coding for homelessness identifies individuals not using formal homeless services and may be useful to support provider efforts to address housing as a health-related social need.

**Supplementary Information:**

The online version contains supplementary material available at 10.1007/s10742-025-00360-x.

## Introduction

On a night in January 2023, a nationwide “point in time” count found over 600,000 individuals were homeless (de Sousa et al. 2023). People experiencing homelessness (PEH) demonstrate significant health disparities compared to housed people (Committee on an Evaluation of Permanent Supportive Housing Programs for Homeless Individuals et al., 2018), and are at a markedly higher health risk, with some estimates suggesting they die, on average, 30 years earlier than their peers with stable housing (Hwang et al. 1997). PEH also have a deep socioeconomic disadvantage and an elevated risk of infectious disease, mental illness, substance use, traumatic injury, and other serious conditions (Aldridge et al. 2018).

Generally, the full population of PEH is not well defined, often relying on the single-night Point in Time estimate referenced earlier. Recent analyses with census data have suggested there are approximately 400,000 PEH, with 200,000 sleeping on the street on a given night (Meyer et al. 2023). National prevalence estimates of the health needs of PEH use epidemiologic data not specific to homelessness (Tsai 2018), outdated data from a nationally representative survey among youth (Ringwalt et al. 1998) or adults (Link et al. 1995), or surveys specific to veterans (Tsai et al. 2016). Estimates of the overall prevalence of homelessness derived from each of these survey studies vary widely, suggesting 4.2–14% of respondents have experienced homelessness sometime in their lifetime and 1.5–4.5% experienced it in the year prior to the survey (Link et al. 1995; Tsai 2018). In healthcare data there are multiple means of examining homelessness, including administrative data linkages (Jacobson et al. 2019), health-system specific EMR flags (Doran et al. 2013), indicators based on addresses (or lack thereof) for payers (*Healthcare Cost and Utilization Project (HCUP) SEDD Notes*, n.d.), or the International Classification of Diseases, 10th Revision (ICD-10) code for homelessness, Z59.0 (Enich et al. 2025; Rollings et al. 2022).

Homelessness is one of many social determinants (or social drivers) of health (SDOH) (Healthy People 2030; n.d.). These broad factors that affect the health of all individuals are increasingly differentiated from health-related social needs, or social and economic needs that are associated with negative health outcomes (U.S. Department of Health and Human Services (HHS), 2023). Health systems and hospitals are more and more interested in and experiencing increased pressure to systematically document health-related social needs– however there is no national noteworthy comprehensive strategy to do so (Centers for Medicare & Medicaid Services, 2024; Friedman and Banegas 2018; Gottlieb et al. 2016).

Adopted widely in the United States in 2015, the ICD-10 introduced Z codes to identify “factors influencing health status and contact with health services”. These codes indicate a wide variety of health circumstances including types of healthcare encounters, disease histories, and a set of ten parent codes meant to identify “persons with potential health hazards related to socioeconomic and psychosocial circumstances,” Z55–65 (Centers for Medicare & Medicaid Services & National Center for Health Statistics, 2024). ICD-10 codes are integrated into electronic medical records for billing, meaning they are a ubiquitously present opportunity to document SDOH; however, Z codes are not consistently understood as mechanisms for documenting screening (Enich and Tiderington 2025; Kepper et al. 2022), even though many medical-record based screeners are relying on them with unknown validity (Cook et al. 2022; Freij et al. 2019).

Among those codes is one specific to homelessness, Z59.0, intended to highlight health risks for homeless patients (Endorf and Nygaard 2021; Weeks et al. 2020). Further, this code can now be specified to Z59.01 and Z59.02, indicating sheltered and unsheltered homelessness, respectively. This is a limited understanding of homelessness, and an illustration of broader challenges in defining homelessness at the federal level, with different total numbers of homeless individuals identified depending on which federal agency’s definition is being used (Sullivan 2023). Most often, homelessness typologies follow the Department of Housing and Urban Development’s homelessness chronicity delineations, identifying someone as chronically homeless after experiencing a chronic, disabling condition and an accumulated one year of unsheltered homelessness over a total of 3 years (Department of Housing and Urban Development 2019). The Homeless Management Information System, or HMIS, is a federally-mandated electronic system meant to document individual’s service use and track what types of services individuals use across the service system (*HMIS: Homeless Management Information System - HUD Exchange*, n.d.); however, research examining HMIS data has shown discrepancies between derived chronic homelessness based on those criteria and being flagged as chronically homeless in HMIS (Cantor et al. 2020).

Despite the unknown validity of its use, Z59.0 was at one point the most commonly used Z code indicator on Medicare claims (Mathew et al. 2020). Research suggests they are underutilized, however (Bensken 2021; Guo et al. 2020; Thompson et al. 2019a, b; Torres et al. 2017; Weeks et al. 2020), and their accuracy in identifying PEH is unknown (Friedman and Banegas 2018; Gottlieb et al. 2016; National Health Care for the Homeless Council 2016). Work examining this code suggests it is neither sensitive nor specific for capturing homelessness, but could have use in ruling in homelessness or capturing PEH not touched by current homeless service systems (Enich et al. 2025; O’Brien et al. 2024; Richard et al. 2019). It also may be more accurate when combined with other data sources, such as self-reported housing instability, or linked to data sets or clinician evaluation (Byrne and Tsai 2022; O’Brien et al. 2024).

Developing an understanding of how Z codes reflect homelessness has important implications for hospital and health system SDOH screening practice and understanding how or if they reflect prevalence of real-world homelessness and other health-related social needs (Stafford and Wood 2017). Our analysis addresses two research questions: (1) What is the prevalence of the ICD-10 code for homelessness, Z59.0, in Medicaid claims, and (2) What are providers’ explanations for the identified patterns and prevalence of the homelessness Z code?

## Methods

### Mixed methods overview

This study was approved by our university Institutional Review Board. It utilized an explanatory sequential mixed methods approach that initially quantitatively analyzed the use of ICD-10 code for homelessness in a large populations-based Medicaid claims database that is linked to homeless service data. Quantitative analysis informed qualitative interviews, which were conducted with inpatient and emergency department (ED) physicians to understand physicians’ explanations for the patterns and prevalence of Z codes. In order to integrate findings into an explanatory model, this study employed a joint display that visualized both quantitative and qualitative results (Fetters et al. 2013; Guetterman et al. 2015).

### Quantitative population and analysis

This study first used secondary, linked data from the statewide New Jersey Medicaid Management Information System (MMIS) from 2016 and the Homeless Management Information System (HMIS) for 19 of New Jersey’s 21 counties from 2014 to 2016. MMIS includes demographic, utilization, and payment data for all New Jersey Medicaid recipients. HMIS was established by the U.S. Department of Housing and Urban Development (HUD) and records the types of services used by people at risk for or experiencing homelessness (*HMIS: Homeless Management Information System - HUD Exchange*, n.d.).

Our unit of analysis was the hospital inpatient or ED encounter or claim, with patient-level information linked into encounters based on the individual for whom the claim was reported. We focused on claims that included the ICD-10 parent code Z59.0, indicating homelessness (World Health Organization, 2004). Demographic, clinical and utilization data were drawn from Medicaid records. Demographic variables included patient age, race and ethnicity, and Medicaid eligibility category. Clinical characteristics included quartile of Chronic Illness and Disability Payment System (CDPS) score (Kronick et al. 2000), the number of physical health diagnoses, presence of a substance use disorder or mental illness diagnoses which we all derived from Chronic Conditions Data Warehouse (CCW) algorithms (Center for Medicare & Medicaid Services, 2021). Healthcare utilization is measured as annual numbers of ED visits and inpatient hospitalizations.

Utilization of homeless services were identified in linked HMIS data, with one binary indicator of for individuals receiving any homeless service recorded in the HMIS (i.e., indicating linkage to at least one HMIS record) from 2014 to 2016. We also adapted a published typology of homelessness based on service use history using three years (2014–2016) of HMIS data; this study hierarchically classified each person using homelessness services in one of five categories: (A) placed in permanent supportive housing (PSH), (B) chronically homeless (experiencing a sum of one year of homelessness over the course of three years and having a chronic, disabling condition) based on HUD standards (HUD User, n.d.), (C) potentially chronically homeless (i.e., meeting HUD standards but not flagged in HMIS as chronically homeless), (D) at-risk of chronic homelessness (Department of Housing and Urban Development 2019) and (E) all others (Cantor et al. 2020).

Statistics are provided on distribution of demographic, clinical and utilization factors across both inpatient and ED claims, stratified by whether claims were for individuals with linked MMIS-HMIS data and not. The unadjusted number claims with a Z59.0 ICD-10 code, and adjusted number of coded claims per 1000 claims were examined across demographic, clinical, and homelessness service use history variables. Chi-square tests were used to assess whether there was a statistically significant difference between proportion of Z-coded claims for each characteristic. Quantitative analyses that are relevant to the qualitative analysis (described below) are presented here, with further analyses, including sensitivity tests of comparisons of HMIS and MMIS indicators of homeless experiences, reported elsewhere (Enich et al. 2025).

### Qualitative sample and analysis

Because they are responsible for diagnostic coding (including Z-codes), we recruited physicians for interviews (Tseng et al. 2018). Our sample was limited to physicians who were aware of or had used Z codes and worked in the inpatient or ED setting of a facility in the highest quarter of Z-coding hospitals based on total number of Z-coded claims or highest rate of Z-coded claims (per inpatient or ED encounter, per HMIS linked encounter, or per county estimated homeless population) (Monarch Housing Associates 2016). Interview participants were recruited using purposive, snowball sampling (Patton 2020). Participants were offered a $30 gift card for completing the interview.

We used a semi-structured interview guide for physician interviews (which is included in the supplemental appendix). Questions in the guide were derived from existing literature on provider SDOH screening (Chhabra et al. 2019; Dauner and Loomer 2021; Herrera et al. 2019), and the limited previous work examining Z code utilization (Bensken 2021; Kepper et al. 2022; Torres et al. 2017). Selected charts based on quantitative analysis were also presented to participants for comment.

Qualitative data were managed, coded, and analyzed using Dedoose software. Interviews were analyzed via deductive content analysis and cross-case inductive thematic analysis (Miles and Huberman 1994; Patton 2020). It was determined that code saturation had been reached within 9 interviews, in that at that point no further insight was offered on what may drive Z code utilization and the ways it fit into documenting health-related social needs. Meaning saturation occurred around 14 interviews, where each code was determined to have a robust description of nuance from each interview (Hennink et al. 2017).

## Results

### Quantitative results: population description and Z-Coding patterns

Table 1 shows analysis of 724,463 claims, including 108,532 inpatient and 615,931 ED claims for 886,352 Medicaid-enrolled individuals, 43,054 with Linked HMIS-MMIS data. Around 1.1% of hospital encounters were for individuals living in PSH, and an equivalent number were chronically homeless or at risk of chronic homelessness (typology categories B-C in Table 1). Compared to the general adult Medicaid population, hospital encounters by the HMIS-linked population tended were more likely to be for people ages 43 to 59 and more people of Black/non-Hispanic race and ethnicity. In terms of clinical risk, encounters of HMIS-linked individuals were more likely to be for those with higher CDPS risk scores as well as higher chronic physical and behavioral health disease burdens, including SUDs, mental health diagnoses, or SMI.

**Table 1:** Z59.0 coding in emergency department and inpatient claims in New Jersey by HMIS Linkage status in 2016^*^

	Total medicaid claims *n* (% col)	HMIS linked population claims *n* (% col)	Proportion of total linked %	Z59.0-coded claims *n* (% col)	Z59.0 coded claims per 1000 of group
Total	724,463 (100)	97,577	13.5	7653	10.6
Sex					
Male	261,800 (36.1)	41,682 (42.7)	15.9	5301 (69.3)	20.3
Female	462,663 (63.9)	55,895 (57.3)	12.1	2352 (30.7)	5.1
Age group					
Age 18–24	112,710 (15.6)	11,035 (11.3)	9.8	439 (5.7)	3.9
Age 25–42	298,708 (41.2)	41,285 (42.3)	13.8	2944 (38.5)	9.89
Age 43–59	217,467 (30)	36,935 (37.9)	17.0	3523 (46)	16.2
Age *≥* 60	95,578 (13.2)	8,322 (8.5)	8.7	747 (9.8)	7.8
Race/ethnicity					
Non-hispanic/White	277,043 (38.2)	35,801 (36.7)	12.9	3722 (48.6)	13.4
Non-hispanic/Black	263,557 (36.4)	48,410 (49.6)	18.4	2745 (35.9)	10.4
Hispanic	110,443 (15.2)	8995 (9.2)	8.1	842 (11)	7.6
Asian	8493 (1.2)	263 (0.3)	3.1	15 (0.2)	1.77
Other^b^	64,927 (9)	4108 (4.2)	6.3	319 (4.2)	5.07
Medicaid eligibility category					
Aged/blind/disabled	222,093 (30.7)	31,233 (32)	14.1	2418 (31.6)	10.9
ACA expansion	237,690 (32.8)	38,465 (39.4)	16.2	4607 (60.2)	19.4
All other	264,680 (36.5)	27,879 (28.6)	10.5	628 (8.2)	2.37
CDPS risk score quartile					
1 st (low)	182,556 (25.2)	15,864 (16.2)	8.7	383 (5)	2.1
2nd	179,921 (24.8)	21,650 (22.2)	12.0	1480 (19.3)	8.2
3rd	180,872 (25)	28,183 (28.9)	15.6	2358 (30.8)	13.0
4th (high)	181,114 (25)	31,880 (32.7)	17.6	3432 (44.8)	19.0
Number of physical health diagnoses^c^					
None	208,591 (28.8)	22,637 (23.2)	10.9	1391 (18.2)	6.7
1	164,366 (22.7)	22,158 (22.7)	13.5	1658 (21.7)	10.1
2–3	166,360 (22.9)	25,560 (26.2)	15.4	2213 (28.9)	13.3
4 or more	185,146 (25.6)	27,222 (27.9)	14.7	2391 (31.2)	12.9
Substance use diagnosis					
Yes	346,540 (47.8)	71,163 (72.9)	20.5	7320 (95.6)	21.1
No	377,923 (52.2)	26,414 (27.1)	7.0	333 (4.4)	0.9
Mental illness diagnoses					
No mental illness	305,406 (42.2)	24,598 (25.2)	8.1	547 (7.1)	1.8
Severe mental illness	294,918 (40.7)	61,309 (62.8)	20.8	6748 (88.2)	22.9
Other mental illness	124,139 (17.1)	11,670 (12)	9.4	358 (4.7)	2.9
Type of claim					
Emergency department	615,931 (85)	84,711 (86.8)	13.8	3723 (48.6)	6.04
Inpatient	108,532 (15)	12,866 (13.2)	11.9	3930 (51.4)	36.2
HMIS homelessness history typology category (2014–2016)^d^					
Unlinked Population (No Homeless Service Use)	626,886 (87)			3783 (49.4)	6.03
A. PSH-placed population		7728 (7.9)			
B. HUD chronically homeless		5919 (6.1)			
C. Potentially HUD chronically homeless		4773 (4.9)			
D. At risk of chronic homelessness		6799 (7)			
E. All other HMIS linked		72,358 (74.1)			

ACA affordable care act, CDPS chronic illness and disability payment system, HMIS homeless management information system, HUD department of housing and urban development, MMIS medicaid management information system and PSH permanent supportive housing

*Measures of age, sex, race, and ethnicity, and Medicaid eligibility group were derived from MMIS. MMIS diagnostic data on claims were sued calculate each beneficiary’s count of physical chronic conditions, SMI/SUD status, and CDPS risk score. All linkage and Z-coding differences are stat significant at p < 0.001

^a^The number of Medicaid beneficiaries in New Jersey in 2016 included 886,352 individuals, 43,054 of whom were linked with HMIS (i.e., they received homelessness services between 2014 and 2016)

^b^To adequately power analyses, this racial/ethnic category was calculated and collapsed into multiple categories. Its composition included 233 claims for those of unknown race/ethnicity, 1,812 of Native American race/ethnicity, and 62,882 for those of “other” race/ethnicity

^c^Out of 27 nonbehavioral health chronic conditions, based on the Centers for Medicare and Medicaid Services Chronic Condition Warehouse

^d^Uses a previously published typology based on Cantor et al. 2020. These typologies were calculated based on three years of HMIS data from 2014 to 2016. The totals do not equal 100% because they were applied only in the HMIS-linked populations

A total of 7653 (10.6 per 1000) claims were Z-coded. Differences in group comparisons by HMIS linkage status were statistically significant (*p* < 0.001) across Medicaid claims. Rates of Z-coded claims per 1000 were significantly higher for the individuals who were male (20.3), age 43–59 (16.2), Non-Hispanic/White (13.4), in the ACA expansion population (19.4), in the highest CDPS risk quartile (19.0), and for those who had four or more chronic physical conditions (12.9) or were diagnosed with an SUD (21.1) or SMI (22.9). The proportion of Z-coded claims was also higher for the Inpatient claims (36.2) and individuals in homeless typology category B (HUD Chronically Homeless) (98.5).

### Qualitative results

#### Participant sample

Table 2 shows the distribution of the qualitative sample participants (*n* = 18) by physician characteristics, Z code familiarity, and whether they were aware whether their hospital had a SDOH screening program. Participants were recruited from eight hospital systems, with between one and five participants per system. All but one interview was conducted over Zoom; the other was conducted in person. Interviews lasted an average of 53 min. All participants had utilized Z codes and documented health-related social needs in the medical record. Major themes from interviews on quantitative trends are described more below.

**Table 2:** Sample characteristics of physician interview participants at high Z-Coding hospitals in New Jersey

Characteristics	*n*	%
Sex		
Male	8	44%
Female	10	56%
Race		
Asian	3	17%
Black/African-American	2	11%
White	13	72%
Ethnicity		
Non-Hispanic/Latino	18	100%
Have you heard of Z codes?		
Yes	16	89%
No	2	11%
Have you used Z codes?		
Yes	9	50%
No	1	6%
Maybe/I don’t know	8	44%
On clarification, have you used Z codes?*		
Yes	18	100%
Have you documented social determinants of health in the medical record?		
Yes	18	100%
Have you documented homelessness specifically?		
Yes	14	78%
No	4	22%
Is there a hospital-level social determinants of health screening at your hospital?		
Yes	10	56%
No	1	6%
Maybe/I don’t know	7	39%
Specialty		
Internal Medicine	7	39%
Emergency Medicine	5	28%
Psychiatry	3	17%
OB/GYN	2	11%
Family Medicine	1	6%
Hospital system distribution		
System A	1	6%
System B	2	11%
System C	1	6%
System D	2	11%
System E	2	11%
System F	1	6%
System G	5	28%
System H	4	22%

*Interview participants who were unsure of or thought they had not used Z codes on clarification said they had put social determinants of health in the problem list, which in all likelihood used Z codes

#### Z codes were accurately understood as uncommon

Participants correctly suggested that Z codes were uncommon and underutilized. An important point that many physicians made was that it is possible that physicians are aware of a patient’s homeless status and for that knowledge to not make it to a hospital claim. This discrepancy could happen at multiple levels, from a physician not assessing whether patients had health-related social needs, discovering them in history taking and not documenting them, documenting them without Z coding them, or even Z-coding it but hospital billers not submitting it in a final claim:

I know when my patients are homeless. I just don’t stick the Z code in there because I’m not really sure why I would, to be honest… there’s certainly patients who I’m not tagging with Z codes, but my documentation reflects they’re homeless. (992)

#### A few high-coding hospitals emerged but participants could not clearly explain trends

Participants could not give definitive explanations as to why their hospitals were high-coding:

[Laughter] I’m laughing because.. I have no idea.. No one—listen, I will tell you that no one has ever come up to me and said, “You really need to document the Z code about homelessness.” Not one person, ever. Ever! (572)

Participants secondarily suggested possible factors that could contribute to high Z-coding, such as local patient populations, reimbursement structure, individual physician priorities, or available hospital services (such as inpatient psychiatric services).

#### Claim coding by demographics were consistent with what participants imagined, with the exception of black/non-hispanic race/ethnicity

Participants believed Z-coding was mostly consistent with what they understood the homeless population to be. Z codes being more prevalent for males, middle aged people and people enrolled in Medicaid via the ACA expansion was consistent with physician’s understanding of the homeless population. Participants specifically highlighted how this is consistent with the presentation of sex and chronic illness covered within the Medicaid population:

I mean, that’s the population where if you have chronic illness by the time it manifests, it’s in that age group you know, so complicated diagnoses don’t happen for 10 years, so you know, so we see kinda advanced disease by then. (777)

While this was the majority opinion, some were surprised the oldest group did not have more Z codes, given the national trend of increasing prevalence of older adult homelessness (Culhane et al. 2013).

The largest racial/ethnic group in the homeless population in NJ were individuals who are Black/Non-Hispanic, followed by people who identified as Hispanic of any race, then White/Non-Hispanic individuals, then individuals of Asian/Unknown/Other race (Monarch Housing Associates 2022). This is a discrepancy when compared to the Z-coding data. However, most participants said that the identified distribution of Z-Coding by race/ethnicity was consistent with what they expected the racial/ethnic breakdown to be in the local homeless population:

They’re not too far off, the White and the Black, like in terms of actual like frequency there, like that’s not a huge difference. For me I think, and this might be my location’s population, but I think it’s almost even for me, like Black and White patients. (495)

Those who did notice the race/ethnicity discrepancy unfortunately could not provide much idea as to why the disparity exists; one person alluded to potential bias in reporting or asking the homelessness screening questions:

I’m a little bit interested in is… kind of how does this compare for population and looking at the race ethnicity breakdown. That that is surprising to me… I think that for white, non-Hispanic than the other groups is not consistent with the community demographic trends, and I think it goes back to this thing of who, of who are we asking the questions to? (893)

#### Participants highlighted the ways in which chronic illness (especially behavioral health conditions) could create opportunities for Z coding

Chronic illnesses are common among PEH; a chronic, disabling condition (most often a mental health or SUD) is one of the criteria necessary in order to determine whether someone is experiencing formal *chronic* homelessness (Department of Housing and Urban Development 2019). As such, disease burden is higher for PEH; participants believed these Z-codings were consistent with the clinical reality of their homeless patient population. This was highlighted in descriptions of the health needs of PEH:

We know that a lot of our patients who are experiencing homelessness have substance use disorders, and a lot of them have mental health problems as well. So, you know, they need access. They need low-barrier access to those two services in particular. (811)

#### Inpatient Z-coded claims being more prevalent was in line with participant understanding

Inpatient claims being Z-coded more frequently than ED claims was consistent with participant expectations. They attributed this largely to the amount of time and complexity of care that physicians provide in the inpatient setting. Almost every participant suggested ED claims were Z coded less frequently because of the high-paced nature of prioritizing acute concerns over assessing for social needs:

Well, ED providers aren’t trying to be thorough. They’re trying to deal with emergencies… We’re here to deal with emergencies, and anything that’s not an emergency, you know, should be, you know, addressed elsewhere. (992)

Z codes were also understood as a way of documenting the complexity of providing medical care, especially in the face of inpatient discharge planning and reimbursement:

You know if you were to look in that inpatient side, like what are some of the problems you’re dealing with are right? Because sometimes somebody is living somewhere and then they have to get discharged and where they are getting discharged to is not – like where they were living before is not a place for them to be going to get discharged to… (970).

In this instance, this provider was speaking to how they had documented homelessness to account for the complex planning process they had to engage in in order to have a patient discharged— and such complex planning most often happens inpatient, according to participants.

### Differences in Z coding by homeless typology are revealing for medical system understanding of homelessness

#### Providers believed patients who were non-homeless-service utilizers and Z-coded were experiencing homelessness

A question of accuracy emerges when there are claims for patients with a Z code without homeless service use; when asked directly about this, multiple participants suggested that patients with a Z code who have not used homeless services could have been intentionally avoiding homeless services. Participants suggested that yes, these individuals are certainly still being *accurately* coded as homeless:

I would guess [the group who had less Z codes who haven’t used homeless services is] a heterogenous group and there’s different reasons. Maybe some of them were never referred to homeless services. Maybe some of them are ill, have mental illness for example, and can’t effectively utilize it. Maybe some of them are using the hospital system for housing. (992)

They highlighted specifically that it is hard to imagine an incentive to inaccurately Z code individuals as homeless if they did not believe the individual to be homeless.

#### Being enrolled in permanent supportive housing was not understood as being homeless

After explaining that PSH is permanent housing with voluntary support services prioritizing people experiencing chronic homelessness (Committee on an Evaluation of Permanent Supportive Housing Programs for Homeless Individuals et al., 2018), everyone agreed: being enrolled in PSH sounded like a stable housing situation and was not experiencing homelessness:

[PSH] just sounds like that’s not a homeless person to me. Yeah, I don’t think I can beat around that but that’s a housed person, that’s how the system works. So I wouldn’t, you know, Z code them for example. (495)

From this one may note that if a patient in PSH described their housing circumstance to a provider it is likely that provider would not understand it as homeless; therefore, it is unlikely they would Z code it as homeless– despite the extensive homelessness history required to enroll in PSH.

#### Chronic homelessness was the most clearly understood contribution to Z coding

Chronic homelessness is a federally-defined homeless status where an individual must have a cumulative year of shelter or place not fit for human habitation stay and a disabling condition (Department of Housing and Urban Development 2019). Participants had difficulty differentiating between homelessness typologies and the formal temporal or disability distinctions between homelessness groups. Despite this, it was clear that individuals understood homelessness chronicity; people experiencing chronic homelessness having a Z code frequently was consistent with participants “recognizing” the image of homelessness in patient care:

[Chronically homeless patients have the most Z codes] probably because the easiest to recognize. And again, it’s probably the most likely to be documented in the chart. A lot of billing and coding comes down to documentation and so it’s more likely that some of these writings in the chart a patient’s homeless. (481)

### Summary of quantitative and qualitative data integration

A summary of mixed methods data integration is presented in Table 3. This table shows that overall major quantitative findings were consistent with what physicians thought quantitative findings would be; they understood Z codes to be uncommon and this was reflected by their overall prevalence. For the most part, they thought demographic, clinical and homelessness chronicity Z-coding patterns were largely reflective of the homeless patients they provided care for. This table draws out the noteworthy instances out, though, of incongruency between hospital Z-coding levels and race/ethnicity, described in greater detail in their respective qualitative sections.

**Table 3:** Data integration and congruency between NJ medicaid Z-coding data and themes identified by physician interview participants

	Quantitative findings/highest % of each group	Qualitative findings	Congruency between data?	Illustrative Quotes
Overall Prevalence	Z codes were uncommon.	Z codes were accurately understood as uncommon.	Yes	That’s not a surprise, right?.. I’m pretty sure the use of the Z code for homelessness is underutilized and that, as I was saying before, there are some barriers for people to even use some of the resources for homeless, so that’s underutilized. (335)
Hospital Character-istics	Fourteen hospitals emerged as high-coders.	A few high-coding hospitals emerged, but participants could not clearly explain trends.	No	I have no idea… maybe they’re doing something different in the physician training process, but I’ve certainly not come across that … I get billing reports for the emergency room and don’t see Z codes there substantially either, so it’s not happening in that setting to my knowledge, so I’m not sure where it’s coming from. (750)
Demographics	Male Sex	That Z codes were more prevalent for males was consistent with physicians’ understanding of males being more prevalent in the homeless population.	Yes	I think from a gender perspective, male homelessness is usually more of a problem. Females may have better luck trying to find, like, temporary situations with friends or otherwise. I’m sure there are studies about why that is, and that I don’t know offhand. (803)
	Age 43–59	Middle age is when chronic illness is first starting to emerge, and participants suggested this is what drove high Z-coding for this population.	Yes	I mean, that’s the population where if you have chronic illness, by the time it manifests, it’s in that age group, you know, so complicated diagnoses don’t happen for 10 years.. so we see kinda advanced disease by then. (777)
	White/Non-Hispanic Race/Ethnicity	Participants were surprised Black/non-Hispanic race/ethnicity was not the most Z-coded category but could not give a clear explanation for why it was not.	No	I see in the inpatient setting of having homelessness and then White/non-Hispanics. Interesting. You know, I might have suspected more Black/non-Hispanics.. Yeah, that’s the only one that kind of surprises me a little bit. (335)
	ACA Expansion Medicaid Eligibility	That individuals enrolled in Medicaid via the ACA expansion were the most Z-coded was consistent with participants’ expectations, given the low to no income of people experiencing homelessness.	Yes	ACA expansion definitely feels right, ’cause they’re the patients who fall into that donut hole. The donut hole is smaller now, but it’s still there, yeah. (481)
Clinical Character-istics	4th Quartile of CDPS Risk	Chronic illnesses are common among people experiencing homelessness; a chronic, disabling condition is one of the criteria necessary to determine someone is experiencing chronic homelessness.	Yes	I think that is consistent with my gestalt—like, if you were to ask me just broadly, are they—would they have greater disability risk, would they have more chronic conditions, would they have substance use or serious mental illness, would they have been identified inpatient as opposed to emergency department, I think all of those things track with my anecdotal experience. (130)
	2–3 Chronic Physical Conditions		Yes	Well, I think these are all important, you know, markers, because if you have severe mental illness, you can’t keep a job; you’re gonna be homeless. If you have multiple medical problems, you may be a foot amputation, can’t keep a job, you know. If you use substances—I always put substance abuse and mental illness, they sort of overlap. (777)
	SUD Diagnosis	Participants specifically highlighted mental illness and substance use as contributory to Z-coding.	Yes	I mean, it is sort of a little bit tied together in the sense that patients with severe mental illness cannot take their medications. Not taking medications, obviously, will allow those chronic conditions to progress to the point where they will into the fourth core value in terms of disability risk scores, so things are sort of tied there. Substance use is the same things. Many times, patients just don’t take their meds. It’s the substance that they’re most interested in. And I don’t know—maybe 60% of them also have mental health issues. (377)
	Severe Mental Illness		Yes	And then, I think the other big bucket that I haven’t touched on, in terms of the health needs, is really around mental health. And again, kind of thinking about—there are some kind of acute mental health needs that may come up, depending on what is the circumstance or how long that person has been experiencing homelessness. And so somebody that may be newly experiencing homelessness, there are a lot of mental health needs that come up that need to be addressed. (893)
	Inpatient Claim	There were more inpatient Z-coded claims because there are more providers, more time spent with patients, and different documentation/billing priorities for discharge planning.	Yes	It’s the inpatient is documenting this more, and I think part of the reason for that is that the patients experiencing homelessness will contribute to length of stay, and so the hospital will start noting this as a means of attempting to get the hospital day covered by the payers. That is almost certainly the reason for the difference. (300)
Homelessness Typology	Z Coded but no HMIS History	Providers believed non-service utilizers that were Z-coded were experiencing homelessness.	Yes	Why Z codes don’t reflect known homeless-service use? I’m just guessing. Maybe the folks who end up in the hospital are the ones who are more likely to be unable to effectively navigate the system and take advantage of the resources. (992)
	PSH Enrollment	Being enrolled in permanent supportive housing was not understood as being homeless.	Yes	[Being in PSH] to me is, like, it just sounds like that’s not a homeless person to me. Yeah, I don’t think I can beat around that, but that’s a housed person. That’s how the system works. So I wouldn’t think that, you know, Z-code them, for example.. I want to say that they don’t sound homeless, so you’re not going to write “Homeless.” (495)
	Chronic Homeless	Chronic homelessness was the most clearly understood contribution to Z coding.	Yes	Yes, I think that makes more sense, because they will be repeat visitors. Yeah, so you will see them, and you will come to know that they are homeless, or somebody will be like, “Oh yeah, he’s homeless,” and you’ll be like, “Yeah,” but will you code it? Not necessarily. (585)

These two areas of incongruency warrant further investigation; participants being unable to describe why their hospitals were high Z coders is consistent with other work highlighting the discrepancy between SDOH screening, and how Z codes ultimately are not seen as codes to document screening but ultimately serve billing purposes (Enich and Tiderington 2025). One participant highlighted a new initiative being developed at their hospital to more directly link a screening process to billing specifically, where physicians would sign off on identified health-related social needs that would ultimately be added to a hospital claim. However, this had not yet been implemented. Every physician acknowledged assessing social determinants in some way as part of their encounters, even if they didn’t bill for it.

The other discordant result was around race/ethnicity, where physicians were correctly surprised to see more White/Non-Hispanic individuals with Z codes for homelessness than other races, despite the known over-representation of Black/Non-Hispanic individuals in our state’s homeless population. This was also surprising to individuals practicing in areas of the state where there is a known population of undocumented Hispanic individuals who are unstably housed. The idea of ascertainment came up most often, with most participants suggesting it would be impossible to apply a Z code to an individual’s claim without asking them about their housing status, suggesting it is possible physicians were just not assessing Black/Non-Hispanic individuals health-related social needs risks as part of their interactions with them. Given physicians inability to describe screening processes beyond their own individual history taking (as described above), it also suggests a limited ability to speak to what systemic factors may be contributing to this inequity in coding.

## Discussion

Interviews with physicians revealed that overall demographic and clinical patterns in the use of the Z59.0 code was consistent with expectations. Demographic characteristics associated with having a Z code in our study population were consistent with some larger patterns in homelessness, such as men (Henry et al. 2021), and people over 60 representing large shares of PEH (Culhane et al. 2013). Included in the definition of *chronic* homelessness, specifically, is having a disabling condition (HUD User, n.d.), which most often includes SUD and/or SMI (Fazel et al. 2014), which is reflected in our discovered prevalences. Throughout interviews, participants suggested that the interplay between homelessness, substance use, mental illness, and/or physical illness all contribute to hospital presentation and Z coding.

Interview participants largely failed to address differences in Z-coding by race; mixed method analysis showed that Z-coding prevalence between White/Non-Hispanic and Black/Non-Hispanic individuals was identified by interviewers as incongruent with the reality of homelessness. Physicians offered some suggestions that this could be driven by local homeless populations, or potential bias in coding patterns. Addressing this difference is crucial to rectifying the anti-Black history of “colorblind” homelessness policies, despite the known racial disparities in prevalence of PEH (Edwards 2021), along with the legacy of anti-Black racism that contributes to significant health disparities (Fowle 2022; National Academies of Sciences et al. 2017).

In the context of billing changes prioritizing reimbursing complex medical care (Centers for Medicare & Medicaid Services, 2022a), and the increasing pressure to screen for SDOH and address health-related social needs (Centers for Medicare & Medicaid Services, 2022b), participants suggested Z codes could emerge as a combined solution to receive higher reimbursement for the work of addressing (and documenting) these needs in medical care. However, billing and reimbursements have not quite kept up with these potential interests; it’s worth noting that any Z code cannot be a stand-alone diagnosis for a given encounter and should only be included when they are noteworthily clinically relevant (Centers for Medicare & Medicaid Services & National Center for Health Statistics, 2024)– despite simultaneous pushes to increase the overall prevalence of these codes (Centers for Medicare & Medicaid Services, 2022b).

Health systems factors also influenced the prevalence of claims having a Z code, including Z codes being more prevalent in inpatient claims and for individuals enrolled in Medicaid via ACA expansion. Other work has highlighted the important effects that ACA expansion had on allowing PEH to be able to access health services, while also acknowledging that expansion alone is not sufficient to address overall low health resource availability for this population (Bharel et al. 2013). It is also possible that the increased proportion of Z-coded inpatient claims is related to documenting needs in the context of complex discharge planning, reimbursement and high readmission rates for PEH (Doran et al. 2013).

Providers themselves suggested nuances within the homelessness designation not reflected in analysis, such as suggesting those patients with a Z code *are* homeless even if they do not use homeless services, indicating individuals enrolled in PSH are *not* homeless, and that those who had no Z code but have used homeless services could still have their homelessness meaningfully addressed even without Z-coding an encounter. These examples could represent the “hidden homeless” population, which have unique health and social service needs that often go unrecognized by current care systems (Crawley et al. 2013). There are discrepancies between Z coding and HMIS status, and the highlighted deficiencies in identifying effective coding practices at specific hospitals and non-White PEH; however, it seems incorrect to say these codes are wholly inaccurate, especially given the moving target of understanding the whole homeless population at any given time (Sullivan 2023). Importantly, from other analyses we can determine that HMIS alone is insufficient to identifying the entire chronically homeless population (Cantor et al. 2020).

In general, initiatives encouraging social determinants of health screening are highly prevalent and screening is happening (O’Brien 2019). The lingering question, then, is how important it is for physicians to accurately ascertain, document and code homelessness as a Z code. There are some complicating factors; previous work has highlighted patients not wanting to disclose social needs if they aren’t going to be relevant to clinical care (Hamity et al. 2018), and provider ambivalence around screening for things that they cannot address (Garg et al. 2016). Specific to homelessness, physicians being able to ascertain where a patient is on the homelessness chronicity spectrum has unknown utility; it is ultimately probably important that they could recognize chronic homelessness, given the increased health burden experienced by these individuals. On the other hand, individuals enrolled in PSH arguably are *not* homeless, since they are (permanently and supportively) housed. However, the chronic disabling condition that qualified them for PSH no doubt persists into this housing, with some hopeful but no definitive research suggesting PSH may address these disparities (Committee on an Evaluation of Permanent Supportive Housing Programs for Homeless Individuals et al., 2018), potentially arguing for interventions to increase physicians ability to understand the nuances in homelessness.

Specific to the housing Z codes, some literature has suggested that codings for any housing-related social need are associated with higher healthcare utilization and illness burden (Rollings et al. 2022). This analysis suggests that current coding practices require more in-depth exploration to measure their utility for identifying homelessness to potentially prevent these outcomes. It also suggests potential utility to blending multiple indicators of homelessness to identify at-risk populations; physicians could play an important role in identifying individuals with a housing health-related social need like in New York, where New York City Department of Health uses homeless service records and also address-based identifiers, Z codes, and other indicators in order to capture the breadth of service need across the health system (Jacobson et al. 2019).

Beyond helping identify a broader population of homelessness, Z codes could also be an opportunity for hospital systems to develop meaningful partnerships with local communities to address this social problem, even beyond the basic requirement from ACA expansion to implement and conduct community health needs assessments—with multiple initiatives highlighted for their ability to address local PEH’s health and housing needs simultaneously (Sandor et al., n.d.). This is in addition to growing interest of state Medicaid initiatives to streamline social determinants of health assessment and documentation, and these agencies highlighting Z codes as a way to operationalize these interests (Ubri et al. 2022).

This study is not without limitations. Claims are generated as part of billing systems and do not provide a comprehensive picture of health status, disability, or other factors that may affect considerations related to homelessness. Additionally, data are from 2016 and interviews were conducted in 2023; it is possible that practices for screening for social needs at both the hospital and individual level have changed since these claims, and that the scope of Z code use was limited given this is early on in its adoption in 2015. This sequencing issue is especially important to highlight because of coding practice and reimbursement changes since 2016, namely that the that billing changes as of January 2023 will influence Z coding patterns as physicians find ways to document for complexity to increase reimbursement with Z codes (Centers for Medicare & Medicaid Services, 2022a).

Additionally, it’s possible that the measured accuracy of the homelessness Z code would be improved by analyzing its concordance with known homelessness at the person-level (rather than encounter-level, as we did). While this may improve the measured validity of the Z code’s ability to reflect known homeless status, its interpretation would in some ways be less intuitive, given that these codes are applied at the encounter-level by physicians. Understanding concordance at the encounter-level adds an important view of how these codes fit into an SDOH screening process; physicians should, some might argue, be discerning and documenting housing status as part of an encounter– or at least when it presents as clinically relevant, and this analysis suggests that is not a regular part of the process of Z code use. However, person-level analysis may more accurately show well how a health system across time is able to discern a patient’s housing status and could be the subject of further investigation to understand the full utility of the Z59.0 code.

Qualitative research is by design not generalizable; perspectives derived from interviews are that of the selected participants only, and cannot be generalized to their hospital system or physicians in the state of New Jersey. For example, to maximize insights about coding practices, we drew participants from hospitals with the highest number or rate of Z coding. Views of clinicians working in other hospitals may be different. Employing a network-based sample may have introduced bias into the results, in that only physicians at certain types of settings or of specific characteristics may have ended up sharing their opinion as part of the research study.

There is an increasing interest in addressing homelessness in hospital settings. This has taken different forms, from more accurate screening (Doran et al. 2021), housing navigation pilot projects with the support of Medicaid funding (F. Thompson et al. 2019a, b), or even embarking on housing development to increase local affordable housing stock (Abrams 2019). Each of these proposed interventions rely on accurate measures of the local homeless population. While annual counts related to both homeless individuals and shelter beds are available (Henry et al. 2021), there is still no clear understanding of how hospital measurements of the local homeless population match with known homeless service utilization. Z codes, since they are internationally standardized, present themselves as a comprehensive opportunity to document key health-related social needs (like homelessness) across a variety of healthcare settings. This study sets the foundation for future partnerships in that realm, shedding light on potential explanatory patterns in homelessness Z coding to either address discrepancies in local homeless population counts or move forward with shared priorities in terms of both health and homeless needs that are rooted in the processes physicians outlined.

## Supplementary Information

Below is the link to the electronic supplementary material.

Supplementary Material 1

## Data Availability

No datasets were generated or analysed during the current study.
