# Supplementary material for: Physician explanation of Z-coded homelessness in medicaid claims
Source: Health Serv Outcomes Res Methodol. 2025 Sep 15;26(3):368–91. doi: 10.1007/s10742-025-00360-x (PMC13334411; doi:10.1007/s10742-025-00360-x)
Supplement: Supplementary file 1 [file 10742_2025_360_MOESM1_ESM.docx]

**Title:** Physician explanation of Z-Coded Homelessness in Medicaid Claims

Supplemental Appendix

[Selected Charts for Interviews	3](#_Toc177066827)

[Interview Guide	7](#_Toc177066828)

[Quote Table	17](#_Toc177066829)

# Selected Charts for Interviews

# Interview Guide

Frame Questions:

1. How do hospital providers understand the use of Z codes in the context of social determinants of health assessment?
2. What are providers’ explanations for the patterns and prevalence of Z codes in Medicaid claims?

*Introduction:* Thanks for agreeing to be a part of this interview. I would like to ask you about your knowledge of homelessness and your use of psychosocial Z codes as part of your direct practice.

As a reminder, what you tell me is entirely confidential and I am prohibited from sharing information that can identify you. I’ll also audio record this interview. The reason I do this is because if it’s recorded I can focus on talking to you right now, and then later transcribe the interview into text to read and analyze. All identifiers you mention (including your name or hospital setting) will be removed from the final product, including my dissertation and any subsequent publications.

Do you have any questions before we begin?

*Interviewer Instructions: Throughout the interview guide, directions to the interviewer are enclosed in brackets and italicized. Questions to be asked are in regular font. Probes are shown as bullet points with italic text.*

[*START RECORDING*]

**Understanding and assessment of social determinants of health**

I want to understand a bit about your understanding of social determinants of health, and how social determinants of health are handled at your hospital. We’re focusing specifically on inpatient and ED visits.

1. To you, what does “social determinants of health” mean to you?
   - *What effects do social factors have on health?*
2. What social determinants of health do you find to be most important?
   - *Why these particular determinants?*
   - *How severe can these health consequences be for people experiencing these determinants, in your view?*
3. **Do you screen for social determinants of health in your clinical encounters? Why or why not?**
   - *Are there ones you screen for more often than others? Which ones?*
   - *Do you see social determinants of health as part of your job?*
   - *If you do screen, what happens after a person screens positive?*
     i. *Is there particular documentation you do?*
     ii. *What do you do about these positives?*
   - *If you don’t screen, do you find they come up as part of your evaluation?*
     i. *Is there particular documentation you do?*
     ii. *What do you do about these positives?*
4. **Is there a systematic process for social determinants of health screening at your hospital?**
   - *What is that process?*
   - *Who is a part of that process?*
   - *What is the documentation affiliated with that process?*
   - *Are there resources you can refer patients to if they screen positive?*

Next I want to ask some questions specifically related to homelessness screening as it relates to social determinants of health.

5. What do you know about the health needs of homeless people?

- *Are you familiar with the concept of chronic homelessness? Housing instability?*
- *Who makes up “homeless people”?*
- *Why do you think some people are homeless?*

6. Do you ask individuals about their housing status? Why or why not?
   - *What does that process look like in a given clinical encounter?*
   - *What is your way of documenting this, if it comes up OR if you ask about it?*
7. [*Especially if they don’t screen]* What do you do if a patient says they’re homeless?
8. Are there any hospital-level interventions around homelessness/housing that you know of?

**Knowledge or utilization of Z codes**

I want to ask some general questions around your knowledge of Z-codes. [*They should, by the inclusion criteria, know what a Z-code is. If they need a reminder:* Z codes are ICD-10 codes that denote factors influencing health status and contact with health services. They include things like being low income or other psychosocial risks, but also things like blood type or genetic cancer predisposition.]

9. Tell me what you know about Z-codes.
   - *Where did you first hear of them?*
   - *What is your understanding of their purpose?*
10. **Do you use them? Why or why not?** [*It’s worth noting they may use them without knowing, so ask if they input social determinants in the EMR in their assessment and plan (likely background is coded as Z)]*
    - *What Z codes do you find yourself using most often?*
    - *What Z codes do you know exist but not use?*
11. **What role, if any, do Z codes play in your social determinant of health screening?**
    - *Is there an EMR-based screening tool?*
    - *Do you know if it documents as Z codes in the chart?*
    - *Do you search for social determinants as part of the diagnosis-entry fields?*
12. What do you know about the Z code for homelessness, Z59.0?
    - *Have you used it before?*
      i. *If yes, when?*
      ii. *If no, what keeps you from using it?*
    - *Where did you first hear of this code?*

Since Z codes are ICD codes, they are affiliated with the hospital billing process. I would like to know a little bit more about how your hospital handles billing.

13. From your knowledge, how does billing work at this hospital?
    - *What role do physicians play in billing here?*
    - *What oversight do you experience in the application of your billing codes?*
    - *Are there other members of the hospital team, like nurses or social workers, that contribute to patients’ bills?*
    - *What do you see your role as in this hospital’s revenue generating process?*
    - *Is there someone that reaches out to you with billing code concerns? If so, do you know who that person and what their role at this hospital is?*
    - *How important does billing feel at this setting?*
14. What do you know about what codes that you put on the medical record ending on a final hospital claim?
    - *Is there a particular part of the medical record that you know contributed to a patient’s final bill, like admitting H&Ps, progress notes, or discharge summaries?*
    - *Do you know if you Z-coded someone, if it would end up on their final hospital claim?*

**Exploration of identified Z-Coding patterns**

We were able to do some quantitative analysis of Medicaid claims (or hospital bills) that were specifically linked to individuals in the Homeless Management Information System, or the system that homeless service agencies all contribute to. Essentially, this means we can see the (deidentified) hospital visit in 2016 of anyone who used homeless services between 2014 and 2016. We looked at a few questions, including how often people with homelessness histories were Z-coded in ED or inpatient encounters, and we measured the degree to which Z codes reflect “true” homelessness.

*Figures 1 & 2*

Figure 1 shows the proportion of Medicaid claims with a homelessness indication. As you can see, the largest represented group is those claims without a Z code and with individuals who have no known homelessness history. Figure 2 shows the agreement between Z codes and any homeless service use on encounters with any homelessness indicator. This shows us that the smallest represented group tends to be the concordant group, or Z-coded claims for individuals with known homeless service use. The next biggest group for both inpatient and ED claims were for those with a HMIS history who were not Z-coded, and finally, a group of claims where there were people Z-coded with no HMIS history.

15. **What are your impressions of these breakdowns?**
    - *What do you think about the total number of Z codes?*
    - *Why do you think there may be differences between inpatient and emergency department claims?*
    - *Do you believe that these people are “actually” homeless?*
    - *How do you think hospital policies effect this?*
16. **What do you think contributes to “missing” a homeless person?**
17. **What do you think might be contributing to providers Z-coding individuals without a homeless history?**
18. **What do you think contributes to concordance, or Z-coding known homeless individuals?**

*Table 1*

Table one shows Z codes’ ability to predict encounters by known homeless service users. You can think of this as Z codes being a “test” for underlying “condition” of homelessness. As you can see, overall sensitivity (or the % of encounters by known homeless individuals that are Z-coded) is low, as “high” tends to be 60% or greater. It however is highest in inpatient claims. Positive predictive value (PPV) or the % of Z-coded individuals who have a homeless history is also relatively low, but highest in the ED.

19. **What are your impressions of these breakdowns?**

- *What do you think might be contributing to providers Z-coding individuals without a homeless history?*
  - *Do you believe that these people are “actually” homeless?*
  - *How do you think hospital policies effect this?*

20. Why do you think Z codes might be more accurate for inpatient claims?
21. What do you think contributes to a higher proportion of Z-coded claims referring to homeless people in the ED?

*Figure 3*

This figure shows the total number of Z-coded claims per 1,000 encounters. Your hospital is highlighted on the left, with the each quartile of Z-coded claims from across NJ hospitals on the right side.

22. **What do you think contributes to your hospital being placed where it is?**
    - *Are there specific hospital-wide initiatives you’d like to highlight?*
    - *How does this reflect social determinants of health screening at your hospital?*
23. What do you think contributes to the hospital with the highest value on the right? The lowest hospitals on the left?
    - *Are there system differences that may be contributing to these differences?*
    - *What contextual factors might contribute to this?*

*Figures 4,5,6*

I’m going to show you a series of graphs describing the number of Z-coded claims per 1,000 claims of a given characteristic. For some context, numbers around 20 or over are relatively high.

Figures 4 shows the number of Z-coded claims per 1,000 claims for each demographic characteristic. Sex, age group, race/ethnicity are self-explanatory. The final group is how someone is enrolled in Medicaid. Here, the most noteworthy characteristics are male sex, being aged 45-69, and General Assistance (enrolling in the ACA expansion).

24. **What are your impressions of these breakdowns?**
    - *Why do you think males, 45-69 year olds, and ACA expansion folks are most represented?*
    - *Adjusting the ACA enrollment category by population increased its overall prevalence. Why do you think such a large proportion of GA-enrolled folks are Z-coded?*

Figure 5 shows the number of Z-coded claims per 1,000 claims of given clinical characteristics. Here, having a high disability risk score, having a substance use disorder or severe mental illness diagnosis, or inpatient claims all stand out. SUDs & SMIs & IP claim are among the highest prevalence ratios.

25. **What are your impressions of these breakdowns?**
    - *Why do you think high CDPS score, 2-3 chronic conditions, SUD diagnosis, SMI, and inpatient claims each have higher prevalence?*
    - *Adjusting the 2-3 illnesses category by population increased its overall prevalence. Why do you think such a large proportion of individuals with 2-3 conditions are Z coded (as opposed to 4 or more, the majority of the category)*
    - *Inpatient differences in adjusted models?*
26. SUD, SMI, and Inpatient are the highest overall prevalence. Why do you think each of those might be?

Finally, figure 6 shows the number of Z-coded claims per 1,000 claims in homeless service use characteristics. Aside from permanent supportive housing, which is one chronic homelessness intervention, you can think about the other categories from left to right in terms of decreasing homelessness severity. Highest ratio was those who were chronically homeless, but second skips a category and goes to disability and less extensive service use. Non-homeless service utilizers, overall, are also higher than service utilizers.

27. **What are your impressions of these breakdowns?**
    - *Why do you think chronically homeless people are most likely to be Z-coded?*
    - *What do you think contributes to a category being skipped?*
28. What do you think of non-homeless service utilizers being more likely to be Z-coded than service utilizers?
    - *Do you think these individuals are “truly” homeless?*
    - *What might contribute to someone Z-coding someone who isn’t homeless?*

In summary, Z codes do not seem to be an accurate representation of homeless service use history. Their use varies greatly by hospital setting and is potentially dependent on provider or hospital social determinant screenings. That being said, for noted homeless individuals chronic homelessness individuals represent have high prevalence among Z-coded claims. However, non-service utilizers overall have more Z codes. Male sex, ACA expansion enrollees, disability, SUDs, SMIs, inpatient Claims all have a high Z code prevalence.

29. What thoughts or overall impressions emerge after looking at all of this data?
30. What “use” do Z codes have to you, or could they have?
31. **What do you think could be done to improve the overall accuracy and utilization of Z-codes?**
32. What questions do you have after reviewing all of this?

Thanks so much for being willing to participate in this study. Any final thoughts on Z codes, social determinants of health screening, homelessness, and these results?

[*STOP RECORDING*]

What’s a good email address to share your gift card with?

Are there other people in your hospital who you think would be willing to participate?

# Quote Table

| **Z Codes Were Accurately Understood as Uncommon** | I think the Z codes are probably underutilized because of lack of awareness. And I think there’s probably also a lack of infrastructure to support, like, how do you—even if you document it, how do you address it, right? (811)  Your data indicates that Z coding is not a very sensitive detection mechanism for homelessness within the system. I don’t know why. The data suggests that it’s probably invoked when it’s helpful and ignored if it doesn’t contribute to the medical picture. Efforts to change that would require either more physician work or more attention from the billers and reimbursement rates could be raised, commensurate with the amount of effort for the Z codes. Then maybe this would be an intervention that would be good for the hospital’s bottom line. (300)  I think, you know, the . . . counterargument to that—and this is really based on my conversation with somebody from Population Healthcare at [a hospital system]—is, like, well, if we’re not documenting it, then we can’t demonstrate that there’s a problem, right? . . . And so I think . . . one of the reasons he really wants to press for provider documentation of it, and the reason he started screening for social determinants, is he really wants to be able to show that there’s a bigger problem than we realize, or at least have the data, you know, to really demonstrate it, you know? (811)  I know the Z codes. . . . It’s a funny thing; you mentioned them in our previous conversation, and then I looked it up and realized to do code Z codes, but I never thought of them as Z codes as a category—you know, separate. Yeah, so when I . . . enter my bills in [the billing program], we just put in the diagnosis, what we want to bill for, and then the code comes up, and I don’t pay attention if it’s I, G, Z, whatever. (377)  The two biggest things are ascertainment. So is there assessment of that person? So—and I guess about who puts the Z code in, right? So if the provider is the one that’s supposed to put the Z code in, then are they ascertaining whether or not that person is experiencing homelessness? Because either through looking at all the other people that have done that ascertainment, versus doing it directly, on the flipside, if the hospital is the one that’s putting in that, then they may be looking at all of the different data points. . . . But I think there’s still this issue that if a provider does not document it, even if it’s a social worker, case manager, nurse . . . as far as I’m aware, that’s not sufficient to put a diagnosis code down. It does need to be done by a provider. (893) |
| --- | --- |
| **A Few High-Coding Hospitals Emerged but Participants Could Not Clearly Explain Trends** | I mean, I would just guess that [our high Z coding rate is] some combination of higher rates of homelessness in [our community] and probably more people are familiar with homelessness as a problem that needs to be addressed, as poverty is such a major issue in our hospital. (992)  I have no idea. Coming from outside of here, I’m bringing a lot of my prior practice with me, so that may be an outlier, and maybe they’re doing something different in the physician training process, but I’ve certainly not come across that in my experience yet, and in my colleagues, I get billing reports for the emergency room and don’t see Z codes there substantially either, so it’s not happening in that setting to my knowledge, so I’m not sure where it’s coming from. (750)  Our hospital in particular does not have an inpatient psychiatric unit… like if you have bipolar disorder and you’re also an opiate user and you’re also homeless, like you are not getting admitted. You are staying in the emergency department. (620)  I mean, I would just guess that [our high Z-coding rate is] some combination of higher rates of homelessness in [our community] and probably more people are familiar with homelessness as a problem that needs to be addressed, as poverty is such a major issue in our hospital. (992)  We know our population, and I think our population is a little bit interesting because we have this—we have both extremes. We have this, like, very core access to care, like, all social determinants of health, like, “Check, check, check,” and then we have the total opposite. We have these people who are, like, very well educated, like you can equally have the CEO of J&J sitting next to some homeless guy. . . . Yeah, but then again . . . we’re not [another urban hospital]. We’re not doing, like, major outreach into our community, but we do know our community very well. (620) |
| **Claim Coding by Demographics Were Consistent with What Participants Imagined, with the Exception of Black/Non-Hispanic Race/Ethnicity** | I believe that. Yes, the male for sure. Interesting: white/non-Hispanic. I mean, still, minorities are overrepresented here, right? Proportionate, you know. I mean, there’s a heck of a lot more White people in this country than there are anybody else. The age, I think, the age distribution—this is all New Jersey? I guess that sort of older age distribution . . . Yeah, it’s very . . . That’s very, very consistent with what I would expect. Yeah, 100 percent. Age, blind, disabled, yeah. (585)  I think from a gender perspective, male homelessness is usually more of a problem. Females may have better luck trying to find like temporary situations with friends or otherwise. (803)  Examining breakdowns by sex, the prevalence of Z-coded males was consistent with physicians’ understanding of males’ prevalence in the homeless population:  I think from a gender perspective, male homelessness is usually more of a problem. Females may have better luck trying to find, like, temporary situations with friends or otherwise. I’m sure there are studies about why that is, and that I don’t know offhand. Perhaps you will be able to tell me later. (803)  Now, male versus female—that’s kind of interesting. I didn’t think there would be a difference. Yeah, it is pretty pronounced that they are documented. Could this be—I don’t know, maybe women mask this or don’t wanna talk about it as much, or—I don’t know. There’s a lot of women shelters, but there’s also men shelters, so I don’t know. And it could be, you know, related to GYN. (777)  I mean, that’s the population where, if you have chronic illness, by the time it manifests, it’s in that age group, you know, so complicated diagnoses don’t happen for 10 years . . . so we see kinda advanced disease by then. (777)  So I think, to my understanding, there’s been a substantial rise in the over-60 homeless population all over the U.S., and so I’m afraid that that group might actually catch up to your 40-plus situation. . . . Why it’s different from the 40-to-60 range, I don’t know. I don’t know if that’s, like, a reporting thing where people are more willing to say that they know resources are possibly out there, and they want help. That’s, like, one of the primary ways people get flagged. (803)  Most participants said the distribution of Z-coding by race/ethnicity was consistent with what they expected the racial/ethnic breakdown to be in the local homeless population. One said,  They’re not too far off, the White and the Black, like, in terms of actual, like, frequency there—like, that’s not a huge difference. For me, I think—and this might be my location’s population, but I think it’s almost even for me, like, Black and White patients. I do think that some of these other—looking at what’s low, like, Asian, Hispanic homeless patients do seem less common in my memory. (495)  Respondent: In regards to race, ethnicity, I would think that would be more heterogenous, and then the ACA expansion may include more of a focused effort on capturing. That is all I can think of.  Interviewer: In New Jersey, Black, non-Hispanic individuals are overrepresented in the homeless population, and yet here, White people are more likely Z-coded. Any idea on why that might be?  Respondent: No. (750)  I see in the inpatient setting of having homelessness and then White/non-Hispanics. Interesting. You know, I might have suspected more Black/non-Hispanics. . . . Yeah, that’s the only one that kind of surprises me a little bit. (335)  Males just strike me as the veteran homeless population . . . and, like, that same age range. . . . This doesn’t surprise me. Honestly, I feel like our homeless population might steer a little bit towards, like, Black, non-Hispanic, but . . . the rest seems pretty in line with my personal experience. (620)  The only thing that I’m a little bit interested in is . . . how does this compare for population and looking at the race ethnicity breakdown? That is surprising to me. . . . I think that for White, non-Hispanic than the other groups is not consistent with the community demographic trends, and I think it goes back to this thing of . . . who are we asking the questions to? (893)  Hispanic—I don’t know if that’s particularly lower. I personally feel like maybe in [my community], that’s probably higher than it is reflected—substantially higher than reflected—but again, couch-surfing homelessness or, like, porch-surfing homelessness is different than accessible, like, access to resources available. And perhaps there’s also a reporting bias associated with that. You know, people are less willing to report if they are undocumented or they can’t access actual services. I don’t know. (803)  Just anecdotally, it does seem like we see more male homeless patients than female ones, and the age range feels about right, and the ACA expansion definitely feels right, ’cause they’re the patients who fall into that donut hole. The donut hole is smaller now, but it’s still there, yeah. (481)  In terms of the ACA expansion . . . I have a hard time connecting that to my memory or my experience, just because it’s not something that I hold onto in terms of thinking back to my patients. (495)  The ACA expansion definitely feels right, cause they're the patients who fall into that donut hole. The donut hole is smaller now, but it's still there, yeah. (481) |
| **Participants Highlighted the Ways in Which Chronic Illness (Especially Behavioral Health Conditions) Could Create Opportunities for Z Coding** | If they’re staying, they are typically staying for, like, a thing, like they are staying for pneumonia, or they’re staying for whatever. A lot of our . . . hospital in particular does not have an inpatient psychiatric unit, so you are going ED to inpatient-unit transfer, so we are keeping all the dual-diagnosis people. . . . Like if you have bipolar disorder and you’re also an opiate user and you’re also homeless, like, you are not getting admitted. You are staying in the emergency department until either you are no longer a danger to yourself, we have some kind of stable discharge planning, or you get transferred to inpatient psych. (620)  I would say, you know, [I’m] definitely not an expert in terms of, you know, what’s the epidemiology on chronic illness in people experiencing homelessness. Besides having a general gestalt of it being, you know, all of the kind of chronic illnesses that we have in our general population being further amplified in terms of heart disease, hypertension, diabetes, renal disease. (893)  Well, I think these are all important, you know, markers, because if you have severe mental illness, you can’t keep a job; you’re gonna be homeless. If you have multiple medical problems, you may be a foot amputation, can’t keep a job, you know. If you use substances—I always put substance abuse and mental illness, they sort of overlap. (777)  I think the other big bucket that I haven’t touched on, in terms of the health needs, is really around mental health. And again, kind of thinking about—there are some kind of acute mental health needs that may come up, depending on what is the circumstance or how long that person has been experiencing homelessness. And so somebody that may be newly experiencing homelessness, there are a lot of mental health needs that come up that need to be addressed. (893) |
| **Inpatient Z-Coded Claims Being More Prevalent as in Line with Participant Understanding** | Interviewer: And what do you think . . . contributes to a higher accuracy in inpatient claims than in the emergency department?  Respondent: Oh. That’s a good question. Probably just more time with the patient, and different people actually looking at the patient, and, you know, having a social worker or transitional navigator, you know, who has to come by the bedside and facilitate—facilitate might not be the best word, but kind of, like, work on discharge planning and bringing issues to the primary treating team that they might not have been aware of and, you know, some other stuff like that. (811)  I think it makes sense that inpatient admissions has more Z coding because I think that it is a more head-to-tail trying to like cover people’s problems and not focus on the presenting problem. (495)  I think the other big bucket that I haven’t touched on in terms of the health needs, is really around mental health. And again, kind of thinking about, there are some kind of acute mental health needs that may come up, depending on what is the circumstance, or how long that person has been experiencing homelessness. And so, somebody that may be newly experiencing homelessness, there are a lot of mental health needs that come up that need to be addressed. (893)  I think there are probably more resources dedicated [to inpatients], so your case manager really will come talk to you at least once those four days, versus in the ED, where you want them in and out, and you have one case manager for, like, your 70 patients that are down there, so I think it’s just harder to data-capture that. Even though you know every time—well, not every time, but a lot of the times, with patients who are homeless or not, they are just not getting documented. (803)  You have more time with the patient [inpatient] than in the ER, because in the ER, you’re maybe a few hours, at most. In the inpatient setting, you have a day, at least, so you have more time to talk with the person. You have more touches, you know, more minutes. (572)  My guess, the reason why [Z-coding] is more in the inpatient side is that those charts are probably looked over more for billing by people who are submitting the billing because, again, the stay is longer. The amount of money is more. So they’re probably going through to pick up more codes, would be my guess. (481)  On the inpatient side, you see most of the Z codes, and I think it’s because the institution really has to—especially public institutions like ours or safety-net institutions—they really have to showcase the amount of effort that goes, which is really tremendous, in terms of caring for an underserved population for their nonmedical needs, so to speak, you know? So I think that’s why. And they have time to think, right? They have coders go over the charts and send queries to the physicians and such. (377)  So when these have, for whatever reason, in terms that I frequently get my wires crossed on ever since coming across them, like, over a decade ago in epidemiology and health policy courses, but I would have thought that the positive predicted value would have been higher inpatient than in the emergency department. It reflects my larger assumption that there’s just so much more noise in the emergency department setting, literally and figuratively, than there is in the inpatient setting, and so I just assume that for almost anything, the signal-to-noise ratio, which here in my mind can clearly use it interchangeably with positive predicted value. (130)  I think it makes sense that inpatient admissions has more Z-coding because I think that it is a more head-to-tail, trying to, like, cover people’s problems and not focus on the presenting problem. (495)  Well, ED providers aren’t trying to be thorough. They’re trying to deal with emergencies. They have a mindset of “That’s what we’re here for. We’re here to deal with emergencies, and anything that’s not an emergency, you know, should be, you know, addressed elsewhere.” (992)  I mean, I think we’ve talked about it—like, substance abuse and mental health is clearly, you know, a big predictor—I don’t know, like a factor. And inpatient, I would wonder, because a lot of these patients have no medical home, so they get so sick that eventually they need to be hospitalized. Because there’s nothing else, you know; we can’t safely discharge them. As an emergency doctor, I’m not gonna discharge them. I’m going to admit them. (435)  As a physician, I probably have an easier trigger to admit somebody who’s homeless than somebody who’s not, because if I walked into the emergency department and I had a cellulitis, a bad cellulitis, right, they would probably feel pretty comfortable that they could give me an antibiotic and have me follow up with my doctor in two days in the office, and that’s all going to get done. If a patient comes in who’s homeless, you’re going to be more likely to admit them and do a much stronger IV, treatment and that kind of stuff. (481)  You know, if you were to look in that inpatient side, like, what are some of the problems you’re dealing with, right? Because sometimes, somebody is living somewhere, and then they have to get discharged, and where they are getting discharged to is not—like, where they were living before is not a place for them to be going to get discharged to. (970)  It made us go back to the conversation about, you know, the inpatient versus emergency department, and kind of thinking about the different Z codes, Z-coding between those. The only other thing I can think about is that with somebody being on the inpatient and, then again, now trying to think about their disposition and the different conferencing that happens over the several days of the person is being hospitalized—so that may be a place where a person is being, where a Z code may then subsequently come up, because then they’re talking about that person’s homeless status, whereas, I mean, people do get their discharge assessments by the social worker in the emergency room, but sometimes that social work service isn’t available 24/7 for kind of the operating of the emergency department, and so depending on the situation, then that may not necessarily happen. (893) |
| **Differences in Z coding by homeless typology are revealing for medical system understanding of homelessness**  (Or deficiencies in the social determinant of health screening to Z coding process) | Why Z codes don’t reflect known homeless-service use? I’m just guessing. Maybe the folks who end up in the hospital are the ones who are more likely to be unable to effectively navigate the system and take advantage of the resources. (992)  That it’s a primary part of their issue, like, that the patient is insightful enough that they’ve shared this is a major stressor. This is something that the treatment team is focusing on. Either they’re new to the situation or they’ve come into some other barrier to other resources. So as a simply co-occurring factor, I would not guess that it happens all that often, but as a primary focus, that would be, I think, one of the rare times where I would include it as a Z code. (750)  I mean, this underscores that for this particular issue, you know, or if trying to understand health-care utilization and people experiencing homelessness. If one were to rely solely on ICD-10 codes, they would be missing a huge chunk of the population, so this just, like, reinforces, I think, that. (893)  Interviewer: Walking through the groups, what do you think contributes to missing somebody—so not Z coding someone who has used homeless services before?  Respondent: Um, prioritization, like, if you’re trying to run through, like, your list and just get it done, you know that may not be top of mind. You are trying to treat them for their diabetes and just, like, shuffle them off. That’s very easy to do, to miss that. Or if it, like, never comes up, like, you can get through a whole history and then two days later find out that they are homeless or, like, couch surfing, and that’s, you know, not necessarily going to be documented right away. Or maybe the patient is too embarrassed to tell you that they are homeless. (803)  I know when my patients are homeless. I just don’t stick the Z code in there because I’m not really sure why I would, to be honest. I don’t know what the utility of doing so is. So, you know, I’m not the only provider seeing my patients, because I’m a consultant. But I would guess that there are other people who also, like me, don’t really know if they should or that they should or why they should, and so when there’s other things to tag, perhaps they tag other things. Because there’s certainly patients who I’m not tagging with Z codes, but my documentation reflects they’re homeless, and I did identify them as homeless in my documentation. (992)  The ones who are not getting coded, it very well may be either that they’re not telling the doctors so the doctors aren’t documenting it, which is also possible, or that just the coders don’t know that they could code for it. It’s probably a combo of those three. (481)  Well, I think depending on where they are—which hospital, what kind of community—they probably don’t want to tell; they are probably not comfortable in telling the provider that they’re homeless, or, I mean, some of it is what kind of issue they are coming into the ER with, and it’s a very personal situation. I think there are so many reasons why somebody would not tell the doctor whether they are homeless or not. (123)  I mean, there’s a stigma attached to homelessness, and so I would imagine that some of that is people not wanting to disclose. . . . So you’re saying that these are folks who we know that they meet the definition, but they weren’t coded? So it’s still on the physician’s end, right? It’s still on our end that we didn’t code them. I think it’s also—it’s some bias, right? You know, because it’s like, how does this person look? It’s easier for me to imagine that somebody who is a substance use disorder is possibly homeless, you know, than my little old lady who, you know, she might live in senior housing. I mean, there’s the bias and the stigmas there, you know? (970)  Well, I don’t know where they would. I don’t know why they would document it if they weren’t. I don’t, so unless that particular code was really close to something that was more common—but not that common because it’s not being coded that often—I would be surprised if they were documenting homelessness when the person wasn’t homeless, because they’re not. Again, it’s a lot of incentive, and they’re not incentivized to. So unless it was a mistake that was easily produced in a systematic way because of something, like, there’s a similar code that they accidentally clicked, and they accidentally clicked this one instead—but otherwise, I would be surprised. (572)  That’s interesting. Is there a definition of homelessness that is not captured? In other words, I remember having a patient who was sleeping on a couch in a friend’s home for the past six months. That’s technically homeless based on definition, but never used shelter or anything, because he was lucky enough to have a couch. So I think this is maybe where the discrepancy might be. (377)  I think that is consistent with my gestalt—like, if you were to ask me just broadly, are they—would they have greater disability risk, would they have more chronic conditions, would they have substance use or serious mental illness, would they have been identified inpatient as opposed to emergency department? I think all of those things track with my anecdotal experience. (130)  Interviewer: Do you think it could be that you have a baked-in idea of who is homeless? Like you think of a homeless person, and you have a middle-aged guy that appears, and so you’re just more likely to remember that when you’re documenting?  Participant: I don’t know. I think that could be the reason. I don’t believe that’s the only reason, but I think that could be a reason. . . . And you’re more likely to remember it when you’re sitting down and documenting your Z code. (572)  When I look at this again, my first inclination is, physician or clinician, whoever it is who is documenting these, they are biased. . . . I think would be more of a reflection of what kind of bias, perhaps, we are experiencing here and the reporting of it. Unfortunately, the Z scores are almost like a subjective measure, right? Like it’s recency bias. You know, it’s the heuristic of substance use homeless person. . . . So, I think this—yeah, I think it reflects our personal thoughts. (803) |
| **Providers Believed Non-Service Utilizers that were Z-coded were Experiencing Homelessness.** | Interviewer: And finally, what about the people who are Z-coded but haven’t used homeless services? What do you think’s happening there?  Respondent: They’re not getting referred to resources. They’re not being pointed in the right direction.  Interviewer: So do you think—I mean, I . . . sort of go back and forth on—do you think those people are, like, actually homeless?  Respondent: Oh, probably.  Interviewer: Do you think there would be incentive to Z-code somebody who’s homeless when they aren’t?  Respondent: Oh, I mean, I think that would be a very rare occasion. I mean, sure. I mean, people—one, people just make mistakes, right? . . . And if there are repeat encounters and that changes, we know that things carry over in the electronic record, like, forever, right? So a person could’ve become domiciled, but because nobody changed it in their chart, they’re still listed as undomiciled, and so that could just potentially . . . But again, I think that’s going to be infrequently the case. (811)  I would guess [the group who are Z coded who haven’t used homeless services is] a heterogenous group and there’s different reasons. Maybe some of them were never referred to homeless services. Maybe some of them are ill, have mental illness for example, and can’t effectively utilize it. Maybe some of them are using the hospital system for housing. (992)  Well, I don’t know where they would. I don’t know why they would document it if they weren’t. I don’t, so unless that particular code was really close to something that was more common—but not that common because it’s not being coded that often—I would be surprised if they were documenting homelessness when the person wasn’t homeless, because they’re not. Again, it’s a lot of incentive, and they’re not incentivized to. So unless it was a mistake that was easily produced in a systematic way because of something, like, there’s a similar code that they accidentally clicked, and they accidentally clicked this one instead—but otherwise, I would be surprised. (572) |
| **Being Enrolled in Permanent Supportive Housing was not Understood as Being Homeless.** | That to me is, like, it just sounds like that’s not a homeless person to me. Yeah, I don’t think I can beat around that, but that’s a housed person. That’s how the system works. So I wouldn’t think that, you know, Z-code them, for example. . . . I want to say that they don’t sound homeless, so you’re not going to write “Homeless.” (495)  The, I’m not sure what to make of the permanent supportive housing though; does that just, for that one over there, does that mean that they’ve had a Z-code claim in at some point in the past, or like within the last calendar year, or just?  Interviewer: Good question. So it’s in 2016, inn 2016 if they had a Z-coded claim, and then their housing status is determined from 2014-2016. So there could be, there could be some time thing happening there.  Respondent: Oh, okay.  Interviewer: Where at some point they were Z-coded and did not yet meet the criteria for--  Respondent: But, right, but then got into permanent supportive housing. Yeah.  Interviewer: Yeah.  Respondent: We need more permanent supportive housing; that’s my, that’s my takeaway. (811) |
| **Chronic Homelessness was the Most Clearly Understood Contribution** | Interviewer: Any thoughts as to why there’s this gap between folks who are not—there’s a lower proportion of Z-coding among people who are not recognized as chronically homeless by homeless-service providers, and then it jumps back up for individuals who are at risk of chronic homelessness.  Respondent: No, I’m not really sure. That’s a good question. I’m not sure. Let me think on it. (481)  Disability and extensive homeless-service use of not meeting the designation either means that is a good definition or, you know, to understand and explore further that group of individuals since you would not—you would expect, then, it should be the kind of the successive decrease in the claims for those individuals. (893)  Like, if you have disability and extensive homeless-service use—this is the person who’s missing a leg, who, you know . . . is homeless, and maybe you’re less likely to document because you know it. It’s in the chart. It’s labeled. But with disability and less extensive homeless-service use, you’re more likely to have—they’ve sought care less. . . . They’re less connected with care. (572)  I think that 76.9 percent just aren’t meeting—it would be interesting to see over time what percentage of those actually end up being in that second large category. You know, pushing that up, I think that would probably—I would surmise be the case if you follow that cohort over time. I would gather that maybe even more than 50 percent of those would end up meeting the criteria for chronically homeless. (335)  Interviewer: Any idea why you think the chronically homeless folks are the most Z-coded?  Respondent: Probably because the easiest to recognize. And again, it’s probably the most likely to be documented in the chart. A lot of billing and coding comes down to documentation, and so it’s more likely that some of these writings in the chart a patient’s homeless. If they’re somebody who are designated, ’cause there’s going to be a lot more signifiers that they’re homeless, probably. (481)  Interviewer: Does it make sense that increasing chronicity of homelessness is potentially Z-coded more often? That if someone is chronically homeless, they are more likely to be Z-coded?  Respondent: Yes, I think that makes more sense, because they will be repeat visitors. Yeah, so you will see them, and you will come to know that they are homeless, or somebody will be like, “Oh yeah, he’s homeless,” and you’ll be like, “Yeah,” but will you code it? Not necessarily. (585)  The way to determine, like, this chronically homeless . . . criteria—I’m not a fan. . . . It just seems like saying a year . . . to be homeless is, like, a really long time; like, that’s just not good. And I know there’s . . . history behind that—probably not really what we’re here to talk about today—but, like, I’m just not a fan. . . . Somebody’s who’s established to be chronically homeless based on the HUD criteria that they’re gonna have a Z-code claim—like, this all . . . seems to make sense. (811) |
